# Meaningful inclusion of people with dementia in interview research: adopting the “intentional stance”

**DOI:** 10.3389/frdem.2025.1596393

**Published:** 2025-06-05

**Authors:** Emma O’Shea, Suzanne Timmons, Kate Irving

**Affiliations:** ^1^Centre for Gerontology and Rehabilitation, School of Medicine, University College Cork, Cork, Ireland; ^2^School of Nursing, Psychotherapy and Community Health, Dublin City University, Dublin, Ireland

**Keywords:** dementia, interview research, semiotic status, person-centered, intentional stance

## Abstract

Engaging people living with dementia in interview research presents unique ethical, methodological, and practical challenges. In recent years there is an increased recognition of the importance and value of meaningfully including people with dementia in research, and of the epistemic injustice of systematic exclusion. While there are a growing number of research papers suggesting strategies for fostering ethical and meaningful inclusion, this area is still very much in development, theoretically and methodologically. This paper outlines how a theoretical perspective on selfhood in dementia, which incorporates the concept of the “Intentional Stance” (as per Sabat), may be a useful means of reaching people with dementia in a meaningful way via open, curious and personhood-supporting interactions. Embodying the “intentional stance” refers to operating under the assumption that all behavior and interactions do have meaning(s), even if it is not immediately or intuitively evident to the researcher what the meaning(s) are. Here, we draw on excerpts from an interview I conducted with a person living with dementia about his experiences of and perspectives on respite and day services, using the intentional stance, in conjunction with a range of other strategies for maximizing reciprocal communication. The analysis highlights instances where the intentional stance was central to connecting with the person, and temporarily entering their lifeworld. Adopting this stance is a means of reducing the epistemic injustice that people with dementia have faced, through longstanding omission and exclusion from research, and from social spheres more broadly.

## Introduction

Dementia is considered a worldwide public health challenge, affecting approximately 55 million people globally, with prevalence projected to rise further in coming decades (World Health Organization, 2023). Historically, research in dementia has been conducted through a biomedical lens. Given the deficit-focus of the biomedical model, the perspectives of healthcare professionals and/or family carers of people with dementia were prioritized as holding more validity than the perspectives of those living with dementia (Niner et al., 2023; O’Shea et al., 2017). This has limited scopes of research inquiry, potentially leading to the proliferation of practices and policies that have failed to reflect the priorities and needs of people with dementia (Rivett, 2017). It constitutes what Halonen et al. (2024) and others (Price and Hill, 2021; Spencer, 2023; Fricker, 2007) refer to as “epistemic injustice,” i.e., mistreating people in their capacity as “knowers,” based on prejudices or stigmatizing attitudes.

In recent years, there has been growing recognition of the value of inclusive research approaches that actively involve people with dementia as research participants and co-researchers, rather than as “subjects” (McConnell et al., 2019; Shannon et al., 2021). However, engaging people living with dementia in research presents unique ethical, methodological, and practical challenges. Cognitive impairments associated with dementia can confound communication, but they do not necessarily preclude participation; rather, they underscore the need for innovative, flexible, and ethically-sound strategies to support inclusion.

There is a growing body of research exploring how we can include people with dementia in interview-based research, focusing on overcoming barriers, and maximizing their ability to contribute meaningfully (Hellström et al., 2007; Murphy et al., 2015; Clarke and Keady, 2002). Many of the strategies noted in these studies have been useful for me, as an early career researcher, who has endeavored to foster inclusive practices. Additionally, guidance from advocacy groups (e.g., Dementia Engagement and Empowerment Project (DEEP)) offers practical tips for researchers on how to foster an ethical and meaningful research relationship/encounter. Some of the common research and advocacy insights mentioned in these works include speaking with family about the person in advance, simplifying language and syntax, asking one question at a time, ensuring “dementia-friendly” environments and privacy, using formal communication aids (e.g., Talking Mats; Murphy and Oliver, 2013), paying attention to non-verbal communication, actively listening, and leading with empathy. Such strategies are sensible and vital elements in any qualitative researcher’s “toolkit.” However, such a toolkit, while necessary, is not always sufficient for facilitating meaningful engagement.

Below, I will outline how a theoretical perspective on selfhood, which can be supported by adopting what Professor Steven Sabat has referred to as the “Intentional Stance” (Sabat and Harré, 1994, p. 147), can create an internal shift in the researcher that can transform the research interaction into something both parties experience as meaningful, while also producing relevant data.

## Selfhood and the intentional stance

A headlining point within Sabat’s philosophy of dementia care is the notion that the effects of dementia can be either aggravated or ameliorated to various degrees, by the way that the person with dementia is positioned by others. This is an uncontroversial take, and is in line with the principles of person-centered care (Kitwood, 1997). However, it was Sabat’s teachings on the “how to” of supporting personhood and selfhood that has had the biggest hand in shaping my research approach. This refers to interrogating your own assumptions about the “semiotic status” of people with dementia.

In order to support personhood, Sabat indicates the value of adopting the “intentional stance” when engaging with people with dementia (Sabat and Harré, 1994). The “intentional stance” is a concept that Sabat adapted from the writings of Dennett (1987). In Sabat’s use, he is referring to the criticality of the assumption that the behavior of people with dementia is *meaning-driven*. He suggests that positionings of people with dementia, underpinned by the core assumption that they are “semiotic beings,” ultimately serves to scaffold their sense of self (Sabat and Harré, 1992; Sabat, 2001).

Sabat critiques the widespread, but often unconscious, tendency to view individuals with dementia as lacking intentionality, particularly when they present with recall and/or communication difficulties. What is really happening, he contends, is that these issues create challenges for the researcher in interpreting meaning; not that there is no coherent meaning to be made. Thus, the key point here, in the context of interview research, is that researchers should conduct interviews under the assumption that all behavior and interactions do have meaning, even if the meaning is not immediately evident to the researcher, i.e., taking an intentional stance.

Sabat and Harré (1994), p.147) elaborate on their concept of “semiotic beings,” defining them as:

“People who can act intentionally in the light of their interpretations of the situations in which they find themselves, and who are capable of evaluating their actions, and those of others, according to public standards of propriety and rationality”

The authors added the following clarification:

“It does not follow that the capabilities will always be realized in speech and action.”

However, they argue that *“creating an appropriate conversational context can make possible the discursive recovery of the power to present oneself as a semiotic subject.”* This is something that I have found to have validity. I will elaborate on this below.

## The intentional stance in interview research

In a research context, we necessarily enter into interview sessions with an agenda guiding how we interact with participants. However, in adopting an intentional stance, you must be able to hold space for the person, and at times that means suspending your research agenda and topic guide.

Finding the meaning, means intending to enter the person’s lifeworld, in as much as you can, and finding creative and natural ways to relate their experiences and narratives to aspects of your research question. Slowing down the pace, and being comfortable with silence are also necessary techniques. Sabat and Harré (1994) encourage embracing silence; specifically, not jumping in to fill silences when the interviewee pauses or appears to need “communication support.”

In 2019, we published a study with six people living with dementia, exploring their perspectives on respite services (O’Shea et al., 2020). The “approach” outlined indicated the adoption of an “empathetic” approach to interviewing (as per Fontana and Prokos, 2016), with the cited theoretical basis being that of Kitwood (1997) person-centred care. However, this description was simply an indicator of the style of interviewing, from my perspective, and the values that I was trying to embody during data collection. In terms of guiding other researchers on how to meaningfully include people with dementia, that description was accurate, but not particularly instructive.

Here, I will elaborate on how the adoption of the “intentional stance” contributed to meaningful inclusion in research, ultimately yielding valuable insights about the perspectives of the participants with dementia on the research topic (i.e., experiences of day and respite services).

Of course, despite adopting the intentional stance, there were still instances where I failed to find meaning during interviews. Sometimes, after these interviews, in particular during the transcription process, I heard something the interviewee had said differently, and hypothesized (albeit too late) other potential meanings and connections. Here however, I want to make the case for the power of adopting the intentional stance, and the potential breakthroughs that can be made in reciprocal communication, which would not otherwise have been possible.

## Case study: “Professor John”

Below is an excerpt from an interview with a man “John” [pseudonym] that I will describe as a case study. John was a University Professor, which his wife indicated had been a core aspect of his identity. At the time of data collection, John was attending day services twice weekly. According to his wife, he was diagnosed with Alzheimer’s disease 6 years prior to the below encounter.

I had a lengthy discussion with John’s wife about his career, their relationship and family life, his interests/hobbies, some of his behavioral and communication “quirks,” and what was on his (and her) mind currently. I also spoke with day service staff about John, and during the recruitment process, one staff member had indicated to me that while John wanted to participate, she did not believe it would benefit my research. She said he often did not make sense, was demanding, and that he sometimes believed he was still working as a Professor. She also noted that he could get quite agitated, and on occasion, physically aggressive.

I proceeded with the interview, since John was eager, and his wife was supportive.

### Interview excerpt 1

I focused the initial stages of the interview on his career. He echoed some of the career highlights his wife had disclosed, and more. At one point, he mentioned how ‘*not all students can be taught marketing methods here*’. I asked him what he meant. The following is the interaction that followed, with some explanatory notes.

John: *“I’ve met some good girls here… Upstanding people, first-class qualifications, MBAs, but they have not a clue what marketing is all about. And the worst thing, they did not seem capable of learning…. [Extended silence] There was no reason given for why we had no choices.”*

I wasn’t sure yet what this meant, if it had relevance to my research question, or where the discussion was going, but I did not need to formulate at this time. I decided to just listen and reflect back my understanding:

“It sounds like they weren’t interested in learning what your needs were?” I asked, tentatively.

John responded: “*No they were not at all interested in learning, in my learning, of my making suggestions… They came to the table with a card and a list of things. Normally they would do 4–5 objectives and my first impressions after 5 or 6 weeks was, ‘is this all that’s on offer?’ There was no meat, no fish, just crisps and potatoes—things I never asked for.*”

I began to suspect that this may be related to the issues with the day service food described by his wife, but I still did not understand this enough from his perspective. I needed to hear more:

I probed: “*This is pretty fresh in your mind?”*

John: “*Very fresh. I began to say at home last night… I asked myself questions… how do I feel about this experience… do I feel rejected? The answer is yes… Do I feel that I did not… that my opinion was not worth taking? The answer is yes… And I did not find it easy to get over it. I’m still suffering inwardly a bit.”*

It was clear that this experience had deeply impacted John. I offered some more space:

EOS: “*From feeling rejected?”*

John: “*Yeah, they raised expectations… I do not know how they did this, but they did not check me out and see what did I want… And yet I knew the girls well and I hated marking them down so much….”*

Here the “*marking them down*” was referenced earlier in another context, and it referred to grading students. In this context, I believed he might be referring to his dissatisfaction regarding staff not enquiring about his food preferences.

I wanted to know if we could anchor his narrative more firmly in relation to the day service. As we were physically in the day service, I asked:

“Would you say that that is typical here [pointing down to the floor]?”

John: “*Yes, I would. In a sense it wasn’t a once-off… The very fact that we had to intervene from Geneva and create a course and a method… What do they like? How do you know they like it? When did you last ask them?”*

The Geneva reference may seem out of place, but it is important and relevant, for John. His wife had disclosed that John had advised international leaders about “economics.” I could never have inferred this otherwise. I reflected this back to him, using his own terminology:

EOS: “*Right – you have to ask the consumers, to get to know the market*?”

It seems he felt seen:

John: “*Oh yes [long pause]… My wife must have known how disappointed I was, if she told you all about this*.”

I tried to both validate this and steer us back to the issue at hand, from my perspective, i.e., lack of choice.

EOS: “*She had mentioned you were not impressed with the food situation here*.”

He elaborated and provided even greater insight into his experience of the power dynamics at play in the service, i.e., the “*hierarchy*.” In particular, he pointed to his feelings of disempowerment, which perhaps were far removed from what his normal was, as a working Professor.

John: “*No… and in fact yesterday, if I had any way of making a decision in that hierarchy, I felt like calling a meeting of everybody, all the teachers and students, and having a general department meeting and asking… Why did they do it*?”

There was a lot within this statement to deconstruct, and so I stayed with this to give more space for him to get his perspective across.

EOS: “*That’s what you felt like doing*?” [long pause].

John: “*Well… Mixed feelings… I am not as satisfied as I was… I was a reasonably satisfied customer 2–3 weeks ago, but since this has happened, I’m not. And if this happens once more, I’m finished… You can be certain there’ll be something signed by me and signed by at least half a dozen others, to say why we are not attending….”*

Acknowledging his desire to take power back, I asked:

“You want to take action?”

He confirmed, nodding, but indicated that trying to foster collective action might be a challenge for him in this context:

John: “*Yes! but the man next to me had the same five chips [that] I had. He did not seem to object! I tried to suss him out, asking ‘are you satisfied?’ I could not draw it out of him.”*

EOS: “*He wasn’t saying either way?”*

John: “*He wasn’t feeling like me, maybe*.”

EOS: “*Hmmm… Maybe… Maybe he wasn’t as vocal about it*?”

John quickly quipped:

“*Maybe I would not be either if I had not been in marketing*.”

We both laughed.

This was a striking display of self-awareness and humor from John, related back to how he had framed much of this discussion, and hit me as an example of how conducting an interview with the intentional stance can bolster selfhood, in a way that allows to you to reach into the person’s lifeworld, and sit in it with them, momentarily.

Of course, the above interaction could have gone entirely differently. I could have pressed on with my interview schedule and hit all the topic points, as I had done many times before.

Instead, I downed tools, listened, and assumed there was meaning in what John was communicating to me. The language at times appeared “irrelevant,” in that the references to “students,” “teachers,” “Geneva,” “departmental meetings,” “creating a course,” and “marking down” could have been interpreted, as the service staff had indicated, as him not always being coherent. In my view, John was simply circumventing word-finding problems he was experiencing, by employing terminology that had been central to his lived experience of complex systems and hierarchical power dynamics. Understanding this, the workaround seemed not only logical, but sophisticated.

### Interview excerpt 2

During the interview, I was curious about John’s thoughts on the activities within the day service. He offered a story, which shone a light on what staff had deemed “aggressive” behavior. While it did not lead to a discussion on activities in the sense that I had envisioned, the resulting conversation was extremely valuable and relevant to my research question.

Me: *Can you tell me about the activities you do here?*

John: *I had some [laughs nervously]… angry activities [Extended silence…] There were two staff one day, who decided to teach me a lesson. I was asking too many questions. And ehm…I could move my seat, they were movable, so I could move it, but not a certain distance because they threatened to block me…*

Me: *Right.*

John: *And that in a sense is threatening to block my ideas…and ehm… and that turned out nasty… I got so annoyed with her…. Do you see this stick here? I used this with both of them [staff members]… I turned it on one of them. I mean I did not ever think it would come to that.*

In this moment, it was clear to me that John was somewhat shocked by his own behavioral reaction. However, he also was also strong in his conviction that his grievances were valid. I leaned into that:

Me: *You were frustrated?*

John: *I was very frustrated… and it was all because of the movement of seats. They wanted to inch me and keep me away from the Headquarters.*

The “Headquarters” reference to me indicated that this was an issue of feeling controlled, in very minor behaviors. John was acutely aware that he was not treated with the same respect he once was. He also understood that his reaction was not acceptable and, in this moment, he chose to gently reassure me that he wasn’t a risk:

John: *And in case you think I’m like that…I would never use it.*

EOS: *I know you will not…*

He continued, describing how jarring the experience was for him:

“I was disappointed in one aspect because there was a girl here that I expected would be on my side and would be open to hearing my views and ehm…strangely enough she didn’t seem to think there was anything wrong with them doing it… They [staff] are mostly nice, but it needs to be sincere.”

Perhaps at the core of his frustration, was that he felt staff *did not* take an intentional stance in this situation, i.e., they did not try to understand why he wanted to move his chair. He felt he had a rapport with one staff member and was thus particularly hurt by her perceived unwillingness to hear his “ideas.” Of course, this interpretation is based on John’s perspective, which while valid, is not a complete account. We say this not because he is living with dementia, but because every person involved in the scenarios he described will have their own perspectives on what occurred, how and why.

## Discussion

The above interaction demonstrates how holding space, learning as much about the person as possible ahead of interactions, and assuming that there is meaning to be made (i.e., adopting the “intentional stance”), will lead to more meaningful engagement, and may lead to fruitful data for answering research questions. These lessons are not just relevant to meaningfully including people with dementia in interview-based research; they are transferrable and apply to communication more generally. They also apply in the context of providing person-centered dementia care. Indeed, we assert that adopting the intentional stance, to support personhood, is a pre-requisite for person-centered dementia care.

While the “intentional stance” might read like a purely academic concept, in practice, it is an internal shift that calls for you to remain open and curious about the person’s experiences and viewpoints. It is a commitment to uphold personhood by trying to understand, regardless of whether you are acquiring the data you set out to collect. Adopting this stance is a means of reducing the epistemic injustice that people with dementia have faced, through longstanding omission and exclusion from research, and from social spheres more broadly.

The use of the “intentional stance” necessitates a level of comfort with making inferences in a way that triangulates various perspectives (in this case, i.e., John’s wife, John, staff members) in real time. A key challenge therein is the question—how much of my interpretation is based upon an appropriate translation of John’s account? Valuable steps toward creating a reflexive space in the research process included: (i) checking my understanding of the interviewee’s experience with them frequently during the interview process, and (ii) interrogating my interview approach and discussing my interpretations of the resulting data with my co-authors. Any use of the intentional stance is fortified by putting in place these safeguards, which helped me to unearth biases and blindspots that one might not otherwise arrive at.

Earlier, I noted how in our 2019 paper (O’Shea et al., 2020), we declared an “empathetic” approach to the interviews with people with dementia, but that this was not particularly methodologically instructive. This is largely because “empathy” as a concept is multidimensional, interpersonal and context-bound and difficult to define (Cuff et al., 2016; De Vignemont and Singer, 2006; Decety, 2020). One way that empathy is commonly understood, is as having both cognitive and affective domains (Cuff et al., 2016), where “cognitive empathy” is the ability to accurately recognize and understand others’ emotional states, while affective empathy refers to the ability to “feel with” others.

In the context of dementia, these cognitive and affective “abilities” relating to empathy are underpinned by assumptions relating to the semiotic status of people with dementia. You must believe that there is intent, and meaning to be made, in order to activate and access genuine empathy, either cognitive or affective for the person you are including in your research.

Thus, purposefully adopting the intentional stance, as was done here with John, can help to bridge the perceived personhood gap that has been created through historical biomedical constructions of dementia. If inclusion in research is to be meaningful, the intentional stance may indeed be a fundamental prerequisite, underpinning interactions with people with dementia. We posit that this stance may be a key means of fostering the type of internal shift needed, to overcome the biomedical construction of dementia that guides many of our unconscious assumptions about the semiotic status of people with dementia. We encourage future research to formally consider the role of the intentional stance when interrogating person-centeredness in the context of research, care, and everyday interactions. Similarly, we encourage health and social care professionals, and the general public, to adopt the intentional stance in their observations of, and interactions with, people living with dementia, and to reflect on the outcomes and implications of those interactions.

## Data Availability

The raw data supporting the conclusions of this article will be made available by the authors, without undue reservation.

## References

[ref1] Clarke C. L. Keady J. (2002). “Getting down to brass tacks: a discussion of data collection with people with dementia” in The perspectives of people with dementia: research methods and motivations. ed. Wilkinson H. (London, UK: Jessica Kingsley Publishers), 25–46.

[ref2] Cuff B. M. Brown S. J. Taylor L. Howat D. J. (2016). Empathy: a review of the concept. Emot. Rev. 8, 144–153. doi: 10.1177/1754073914558466

[ref3] De Vignemont F. Singer T. (2006). The empathic brain: how, when and why? Trends Cogn. Sci. 10, 435–441. doi: 10.1016/j.tics.2006.08.008, PMID: 16949331

[ref4] Decety J. (2020). Empathy in medicine: what it is, and how much we really need it. Am. J. Med. 133, 561–566. doi: 10.1016/j.amjmed.2019.12.012, PMID: 31954114

[ref5] Dennett D. C. (1987). The intentional stance. Cambridge: Bradford Books/MIT Press.

[ref6] Fontana A. Prokos A. H. (2016). The interview: from formal to postmodern. New York: Routledge.

[ref7] Fricker M. (2007). Epistemic injustice: power and the ethics of knowing. Oxford: Oxford University Press.

[ref9001] Halonen U. Aaltonen M. Aerschot L. V. Pirhonen J. (2024). Participation of persons living with dementia in research: A means to address epistemic injustice. Dementia. 14713012241299015. 39510100 10.1177/14713012241299015 PMC12171031

[ref8] Hellström I. Nolan M. Nordenfelt L. Lundh U. (2007). Ethical and methodological issues in interviewing persons with dementia. Nurs. Ethics 14, 608–619. doi: 10.1177/0969733007080206, PMID: 17901172

[ref9] Kitwood T. (1997). Dementia reconsidered: the person comes first. Buckingham: Open University Press.

[ref10] McConnell T. Sturm T. Stevenson M. McCorry N. Donnelly M. Taylor B. J.. (2019). Co-producing a shared understanding and definition of empowerment with people with dementia. Res. Involv. Engag. 5, 1–11. doi: 10.1186/s40900-019-0154-2, PMID: 31205750 PMC6558688

[ref11] Murphy K. Jordan F. Hunter A. Cooney A. Casey D. (2015). Articulating the strategies for maximising the inclusion of people with dementia in qualitative research studies. Dementia 14, 800–824. doi: 10.1177/1471301213512489, PMID: 24403314

[ref12] Murphy J. Oliver T. (2013). The use of talking Mats to support people with dementia and their carers to make decisions together. Health Soc. Care Community 21, 171–180. doi: 10.1111/hsc.12005, PMID: 23095078

[ref13] Niner S. Bran F. Mohan D. Warren N. (2023). Evaluating the extent of participation of people living with dementia in research. Int J Qual Methods 22:16094069231211247. doi: 10.1177/16094069231211247

[ref14] O’Shea E. O’Shea E. Timmons S. Irving K. (2020). The perspectives of people with dementia on day and respite services: a qualitative interview study. Ageing Soc. 40, 2215–2237. doi: 10.1017/S0144686X1900062X

[ref15] O’Shea E. Timmons S. O’Shea E. Fox S. Irving K. (2017). Key stakeholders’ experiences of respite services for people with dementia and their perspectives on respite service development: a qualitative systematic review. BMC Geriatr. 17:282. doi: 10.1186/s12877-017-0676-0, PMID: 29216836 PMC5719558

[ref16] Price K. A. Hill M. R. (2021). The silence of Alzheimer’s disease: stigma, epistemic injustice, and the inequity of those with progressive cognitive impairment. Commun. Res. Pract. 7, 326–343. doi: 10.1080/22041451.2021.2006113

[ref17] Rivett E. (2017). Research involving people with dementia: a literature review. Working Older People 21, 107–114. doi: 10.1108/WWOP-11-2016-0033

[ref18] Sabat S. R. (2001). The experience of alzheimer’s disease: Life through a tangled veil. Oxford: Blackwell.

[ref20] Sabat S. R. Harré R. (1992). The construction and deconstruction of self in alzheimer's disease. Ageing Soc. 12, 443–461. doi: 10.1017/S0144686X00005262

[ref21] Sabat S. R. Harré R. (1994). The Alzheimer's disease sufferer as a semiotic subject. Philos. Psychiatry Psychol. 1, 145–160.

[ref22] Shannon K. Montayre J. Neville S. (2021). Nothing about us without us: research methods enabling participation for aged care residents who have dementia. Int J Qual Methods 20:16094069211055938. doi: 10.1177/16094069211055938

[ref23] Spencer L. (2023). Epistemic injustice in late-stage dementia: a case for non-verbal testimonial injustice. Soc. Epistemol. 37, 62–79. doi: 10.1080/02691728.2022.2103474, PMID: 36816431 PMC9928428

[ref24] World Health Organization (2023). Fact sheets of dementia. Geneva: World Health Organization.

